# Supplementary material for: Validity of the Cancer and Aging Research Group Predictive Tool in Older Japanese Patients
Source: Cancers (Basel). 2022 Apr 21;14(9):2075. doi: 10.3390/cancers14092075 (PMC9104937; doi:10.3390/cancers14092075)
Supplement: Supplementary file 1 [file cancers-14-02075-s001.zip › cancers-1659454-supplementary.pdf]

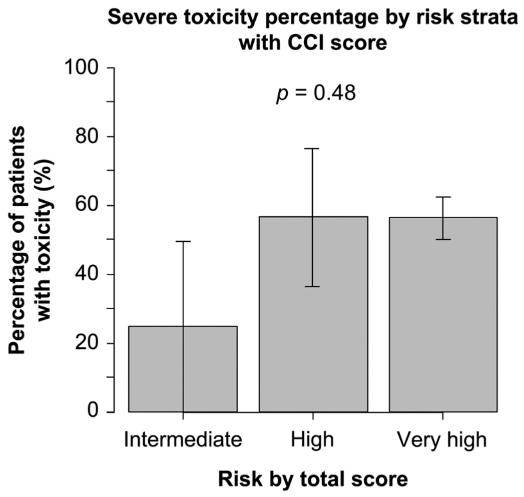


**Figure S1.** Ability of CCI to predict grade 3–5 CRAEs. Abbreviations: CCI, Charlson comorbidity index; CRAEs, chemotherapy-related adverse events.

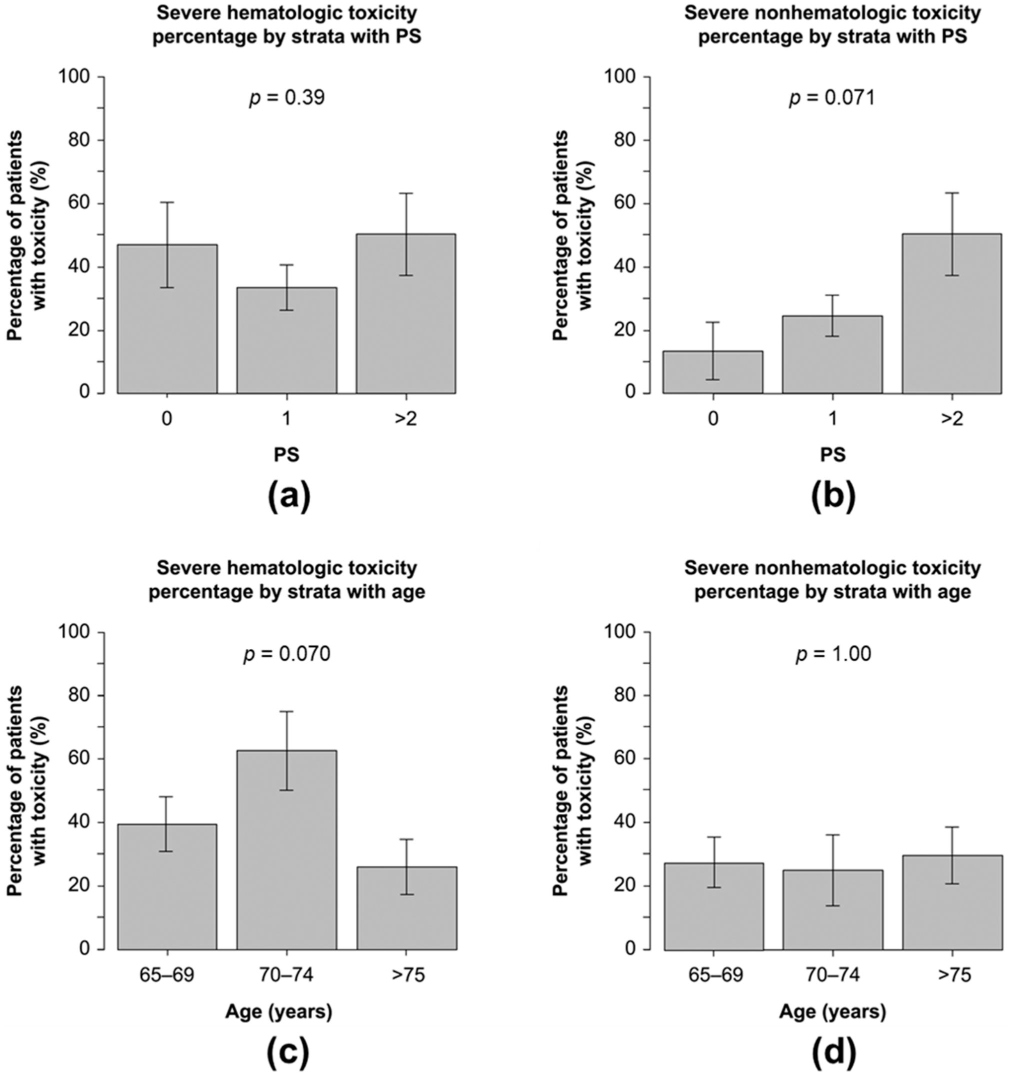


**Figure S2.** The percentage of participants with severe hematologic (a) and nonhematologic (b) toxicity based on ECOG PS scores or severe hematologic (c) and nonhematologic (d) toxicity based on age. Abbreviations: ECOG PS, Eastern Cooperative Oncology Group performance status.

**Table S1.** Prediction model and scoring algorithm for chemotherapy toxicity.

| Variable | Value/Response | Score |
|---|---|---|
| Age (years) | ≥72<br><72 | 2<br>0 |
| Cancer type | GI or GU cancer<br>Other cancer types | 2<br>0 |
| Planned chemotherapy dose | Standard dose<br>Dose reduced upfront | 2<br>0 |
| Planned No. of chemotherapy drugs | Polychemotherapy<br>Monochemotherapy | 2<br>0 |
| Hemoglobin | <11 g/dL (male),<br><10 g/dL (female)<br>≥11 g/dL (male),<br>≥10 g/dL (female) | 3<br>0 |
| Creatinine clearance | <34 mL/min<br>≥34 mL/min | 3<br>0 |
| How is your hearing (with a hearing aid, if needed)? | Fair, poor, or totally deaf<br>Excellent or good | 2<br>0 |
| No. of falls in the past 6 months | ≥1<br>None | 3<br>0 |
| Can you take your own medicine? | With some help/unable without help | 1<br>0 |
| Does your health limit you in walking 100 m? | Somewhat limited/limited a lot<br>Not limited at all | 2<br>0 |
| During the past 4 weeks, how much of the time has your physical health or emotional problems interfered with your social activities (visiting with friends, relatives, etc.)? | Limited some of the time, most of the time, or all of the time<br>Limited none of the time or a little of the time | 1<br>0 |

GI, gastrointestinal; GU, genitourinary.

**Table S2.** Chemotherapy-related adverse events.

| | Grade 3–5 | | Grade 3 | | Grade 4 | |
|---|---|---|---|---|---|---|
| | No. | % | No. | % | No. | % |
| Hematologic | | | | | | |
| Leukopenia | 14 | 18 | 12 | 16 | 2 | 3 |
| Neutropenia | 21 | 28 | 12 | 16 | 9 | 12 |
| Anemia | 11 | 14 | 9 | 12 | 2 | 3 |
| Thrombocytopenia | 2 | 3 | 1 | 1 | 1 | 1 |
| Febrile neutropenia | 3 | 4 | 3 | 4 | 0 | 0 |
| Nonhematologic | | | | | | |
| Fatigue | 12 | 16 | 12 | 16 | 0 | 0 |
| Nausea | 12 | 16 | 12 | 16 | 0 | 0 |
| Mucositis oral | 3 | 4 | 3 | 4 | 0 | 0 |
| Diarrhea | 1 | 1 | 1 | 1 | 0 | 0 |
| Hypertension | 1 | 1 | 1 | 1 | 0 | 0 |
| Proteinuria | 1 | 1 | 1 | 1 | 0 | 0 |
| Edema | 1 | 1 | 1 | 1 | 0 | 0 |
| Hyponatremia | 2 | 3 | 1 | 1 | 1 | 1 |
| Hyperkalemia | 2 | 3 | 2 | 3 | 0 | 0 |
| Hypomagnesemia | 1 | 1 | 0 | 0 | 1 | 1 |
